# Transplanting

**Published:** 1879-08

**Authors:** 


### TRANSPLANTING.

About the twentieth of March, 1879, Master C., aged twelve and a half years, by a fall, broke the left central superior incisor. The fracture began on the anterior surface at the margin of the gum, and extended obliquely across to a point a line and a half above the margin of the gum at the lingual side. There was no inflammation or soreness about the root after the accident. The pulp died within a few days, the debris was removed and the canal thoroughly cleansed. It remained in this condition about three weeks.

The first intention was to adjust a crown by a pivot. There was hesitancy about this course, because of the age of the patient, and because the canal through the root was large, and rather larger as it approached the apex, and because the fractured end did not present a favorable form for the reception of the artificial crown.

It was finally decided to remedy the breach by transplanting so soon as a suitable tooth could be obtained. Within a few days a tooth of about the right size, form and color was presented for extraction, though with a small decay in the anterior proximate surface.

The tooth was removed, the boy sent for, and by the time he arrived the cavity of decay and root canal were filled.

Upon the arrival of the boy the root of his tooth was carefully extracted, and the other at once introduced. It was, upon close inspection, slightly narrower than the right central, but in other respects a perfect match. The root was slightly smaller than the one removed.

It was ligated to the adjoining teeth for two or three days, when it was found to be attached and the ligature was removed.

There was no soreness, and the tooth, within two months, became as strong as its fellows; maintains its color perfectly, and performs its work equal in all respects with the adjoining teeth.

The entire absence of inflammation and soreness, and the rapidity with which union and fixedness were attained is significant and instructive.

